# Rationale and design of a multicenter, double-blinded, randomized, placebo-controlled trial to investigate the effects of naldemedine on opioid-induced constipation for patients with cancer pain: A study protocol

**DOI:** 10.1016/j.conctc.2022.100967

**Published:** 2022-08-05

**Authors:** Takahiro Higashibata, Jun Hamano, Takaomi Kessoku, Shinya Kajiura, Mami Hirakawa, Yoshiki Horie, Masaki Shimizu, Shunsuke Oyamada, Keisuke Ariyoshi, Kota Kihara, Yohei Yamanaka, Kumi Konishi, Kosuke Doki, Yasuyuki Takashima, Manabu Horiuchi, Masato Homma, Takeshi Yamada, Yoshiyuki Yamamoto, Toshikazu Moriwaki, Tatsuya Morita, Atsushi Nakajima, Hiroka Nagaoka

**Affiliations:** a Palliative Care Team, Department of General Medicine and Primary Care, University of Tsukuba Hospital, 2-1-1 Amakubo, Tsukuba, 305-8576, Japan; b Faculty of Medicine, University of Tsukuba, 2-1-1 Amakubo, Tsukuba, 305-8576, Japan; c Department of Palliative Medicine, Yokohama City University Hospital, 3-9 Fukuura, Kanazawa-ku, Yokohama, 236-0004, Japan; d Department of Gastroenterology and Hepatology, Yokohama City University School of Medicine, 3-9 Fukuura, Kanazawa-ku, Yokohama, 236-0004, Japan; e Department of Clinical Oncology, Toyama University Hospital, 2630 Sugitani, Toyama, 930-0194, Japan; f Department of Palliative Medicine, St. Marianna University School of Medicine, 2-16-1 Sugao, Miyamae-ku, Kawasaki, 216-8511, Japan; g Department of Clinical Oncology, St. Marianna University School of Medicine, 2-16-1 Sugao, Miyamae-ku, Kawasaki, 216-8511, Japan; h Department of Palliative Care, Kyoto Katsura Hospital, 17 Yamada Hirao-cho, Nishikyo-ku, Kyoto, 615-8256, Japan; i Japanese Organisation for Research and Treatment of Cancer (JORTC) Data Center, 2-54-6-302 Nishinippori, Arakawa-ku, Tokyo, 116-0013, Japan; j Japanese Organisation for Research and Treatment of Cancer (JORTC) Operations Office, 2-54-6-302 Nishinippori, Arakawa-ku, Tokyo, 116-0013, Japan; k Department of Pharmacy, University of Tsukuba Hospital, 2-1-1 Amakubo, Tsukuba, 305-8576, Japan; l Tsukuba Clinical Research & Development Organization, University of Tsukuba, 2-1-1 Amakubo, Tsukuba, 305-8576, Japan; m Department of Pharmaceutical Sciences, Faculty of Medicine, University of Tsukuba, 2-1-1 Amakubo, Tsukuba, 305-8576, Japan; n Department of Gastroenterology, University of Tsukuba Hospital, 2-1-1 Amakubo, Tsukuba, 305-8576, Japan; o Division of Palliative and Supportive Care, Seirei Mikatahara General Hospital, 3453 Mikatahara-cho, Kita-ku, Hamamatsu, 433-8558, Japan

**Keywords:** Opioid-induced constipation, Naldemedine, Prevention, Randomized controlled trial

## Abstract

**Background:**

It is unclear which laxatives are appropriate to prevent opioid-induced constipation (OIC). This study will evaluate whether prophylactic use of naldemedine prevents OIC in patients with cancer who start opioid administration.

**Methods:**

This study is a multicenter, double-blinded, randomized, placebo-controlled trial. Patients who meet the eligibility criteria and give consent will be randomly assigned to the naldemedine or placebo group. Both groups will take each drug once a day after breakfast for 14 days.

**Results:**

The primary endpoint is the proportion of patients with a Bowel Function Index of less than 28.8 on Day 14. The secondary endpoints include assessment scales of the impact of constipation on comprehensive quality of life.

**Conclusions:**

This is the first study proposed to assess the superiority of naldemedine over placebo in the prevention of OIC. If naldemedine is found to be effective in reducing OIC compared with the placebo, it will be regarded as a new standard for OIC prophylaxis at opioid initiation.

**Trial registration:**

jRCT identifier: jRCTs031200397. Registered March 5, 2021, https://rctportal.niph.go.jp/en/detail?trial_id=jRCTs031200397.

## 1 Background

Constipation occurs in 63–81% of patients with cancer receiving opioids [1] and is likely to occur early in the course of opioid therapy [2]. Therefore, careful monitoring and usage of laxatives to prevent constipation are recommended by national guidelines. Currently, osmotic laxatives and colon-stimulating laxatives are commonly used for the treatment of opioid-induced constipation (OIC) [3]. In addition, patients with cancer who receive laxatives at the time of opioid initiation often have less OIC [4]. The guidelines of the American Gastroenterological Association and the Japan Society of Palliative Medicine recommend the use of laxatives to prevent constipation when opioids are administered [5,6]. However, it is unclear which laxatives are appropriate to prevent OIC.

Naldemedine is a peripherally-acting μ-opioid receptor antagonist (PAMORA) that specifically relieves OIC [7]. In patients with cancer, a secondary analysis of a comparative study with pain as the primary endpoint in patients initiating opioids for cancer pain demonstrated slightly less constipation in the group of patients who received PAMORA [8]. However, there is insufficient evidence regarding the superiority of PAMORA over placebo for the prevention of OIC, which is an issue to be addressed.

Therefore, this study will evaluate whether the prophylactic use of naldemedine prevents OIC in patients with cancer who start opioid administration compared with placebo. We hypothesize that naldemedine can prevent the development of OIC if it is taken from the start of regular strong opioid medication. If naldemedine is found to be effective in reducing OIC compared with placebo, it will be regarded as a new standard for OIC prophylaxis to be administered concurrently at opioid initiation.

## 2 Methods

### 2.1 Study design and setting

The Standard Protocol Items for Randomized Trials statement and its checklist were followed in preparing the study protocol. This study was designed to be a double-blinded, placebo-controlled trial. It will be conducted in the University of Tsukuba Hospital, the Yokohama City University Hospital, the Toyama University Hospital, and the St. Marianna University School of Medicine. In this study, the electronic data capturing (EDC) system will be used to collect data. The patient diary, which includes patient-reported outcomes, will be provided in paper form and collected at each institution.

### 2.2 Ethical considerations and registration

This study will be conducted in accordance with the Declaration of Helsinki and Japan's Clinical Trials Act. The protocol was approved by the Tsukuba University Clinical Research Review Board on January 26, 2021 (approval reference number TCRB20-001). Prior to the start of patient enrollment, the Research Office registered the study in the Japan Registry of Clinical Trials (jRCT) as jRCTs031200397. When changes are made to the protocol, an “Application for Protocol Revision” must be submitted to the secretariat of the Independent Data Monitoring Committee. A protocol change is defined as one or more of the following: i) may increase the risk to patients participating in the trial, ii) substantially affects the primary endpoint, or iii) essentially affects the conduct of the trial. After the protocol revision is approved by the Independent Data Monitoring Committee, the principal investigator shall submit an application for protocol modification to the Accredited Clinical Research Review Board.

Prior to enrollment, the principal investigator or sub-investigator should provide the patient with an explanatory document approved by the Accredited Clinical Research Review Board. If the patient agrees to participate in the study, the patient's signature will be obtained using the consent form. We recognize that personal information, medical information, and other information related to privacy should be strictly protected and handled carefully under the principle of respecting the individual's details. We will take the necessary management measures to protect privacy. Of the personalized materials of registered patients related to this study, the original materials used for clinical research (consent forms, medical records used as the source of Case Report Forms, and patient diaries) must be retained for at least five years after the end of the study. If they are to be destroyed after that, appropriate measures (deletion of data, shredding of paper, etc.) must be taken such that specific individuals cannot be identified. Regarding health damage caused by participation in this clinical trial, appropriate treatment according to the medical condition will be provided as insurance-covered treatment in the same manner as standard medical treatment. In this case, the patient will be responsible for out-of-pocket medical expenses.

### 2.3 Intervention

**Fig. 1:**
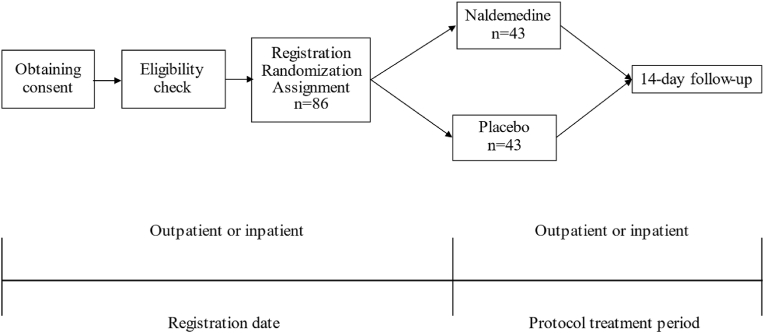
Study flow.

Patients who meet the patient eligibility criteria and give consent will be randomly assigned to the naldemedine (Symproic® 0.2 mg) or placebo group (Fig. 1). The protocol treatment period will be 14 days after the start of naldemedine (or placebo) and the naldemedine group will take Symproic® at 0.2 mg once a day after breakfast for 14 days. The placebo group will take the placebo once a day after breakfast for 14 days. The first dose of naldemedine or placebo will be given orally with the first oral administration of strong opioids, not just after breakfast. No change in the administration method during the protocol treatment period is allowed, but if it is judged to be medically dangerous, the treatment should be changed according to the medical judgment of the physician in charge. This will be considered a protocol deviation, but if it is judged to be medically appropriate, it will be considered a clinically appropriate deviation and included in the analysis population.

Regular laxatives used prior to registration will be continued without change until the end of the protocol treatment. However, if there is a concern about a decrease in the quality of life (QOL) (e.g., diarrhea), a reduction or interruption of the regular dose of laxatives will be allowed. Except for regular laxatives used prior to enrollment, no regular laxatives will be used during the protocol treatment period. Rescue laxatives will be prescribed at the time of enrollment regardless of whether regularly administered laxatives are used. However, rescue laxatives may be used only if the patient has not had a bowel movement for more than two days. For rescue laxatives, two sennosides will be the first choice based on the clinical situation at the participating sites.

### 2.4 Outcome measurements

The primary endpoint will be the proportion of patients with a Bowel Function Index (BFI) of less than 28.8 on Day 14. The BFI is a numerical rating scale that evaluates the ease of defecation, residual stool, and symptoms of constipation over the previous seven days, and the average numerical rating scale of the three items of 28.8 or higher is defined as OIC [9,10]. Although this scale requires confirmation by researchers, a health care provider (trained physician, nurse, or pharmacist) who is not aware of the allocation results will check for omissions or errors in the scale in person, by telephone, or by online interview in order to ensure blinding.

The secondary endpoints will include (1) other measures of the effects of naldemedine on the development of OIC, proportion of patients with a BFI of less than 28.8 on Day 7, amount and rate of change from Day 1 in BFI for Days 7 and 14, proportion of patients with 3 or more spontaneous bowel movements (the number of defecations, excluding those within 24 h after rescue laxative administration [7,11]) per week on Days 7 and 14 (frequency), proportion and number of patients with 3 or more complete spontaneous bowel movements (the number of bowel movements without a residual stool feeling, excluding bowel movements within 24 h after rescue laxatives were administered [7,11]) on Days 7 and 14, bowel movement during defecation (yes/no), squeezing during each defecation (not at all/just a little/moderately/quite a lot/very much), residual feeling during each defecation (yes/no), and overall bowel movements (4 levels: dissatisfied, somewhat dissatisfied, somewhat satisfied, and satisfied) in the last week [12] on Days 1, 7, and 14, and changes in bowel movements between Days 1 and 7 and between Days 1 and 14, (2) assessment of the impact of constipation on comprehensive QOL; the Japanese version of the Patient Assessment of Constipation Quality of Life questionnaire (PAC-QOL) [13,14] and the Patient Assessment of Constipation Symptoms questionnaire (PAC-SYM) [15] on Days 1 and 14, European Organization for Research and Treatment of Cancer Quality of Life Questionnaire Core 15 Palliative (EORTC QLQ-C15-PAL) [[16], [17], [18]] and subscale scores for Days 1, 7, and 14, proportion of patients who responded to EORTC QLQ-C15-PAL item 10 (constipation) on Days 1, 7, and 14, amount of change in PAC-QOL on Days 1 and 14, and amount of change in PAC-SYM on Days 1 and 14, (3) effects of naldemedine on the development of opioid-induced nausea and vomiting; proportion of patients who had at least one episode of vomiting during the 72-h period from Days 1–3, proportion of patients who used antiemetic drugs during the 72-h period from Days 1–3, and proportion of patients who responded to EORTC QLQ-C15-PAL item 9 (nausea) on Days 1, 7, and 14, (4) number of times rescue laxatives were used, (5) number of diarrhea episodes that occurred during administration of the study drug (Diarrhea is defined as Bristol Stool Scale type 6 or 7 [19], in accordance with a previous study [20].), (6) adverse events that occurred during study drug administration, and (7) double-blind validation (Table 1).

**Table 1:** Study schedules of observations, tests, and assessments.

	Before registration	Protocol treatment period													
	Visit	Day	Day	Day	Day	Day	Day	Day	Day	Day	Day	Day	Day	Day	Day
Item	1	1	2	3	4	5	6	7	8	9	10	11	12	13	14
Obtaining consent	●														
Registration eligibility verification	●														
Patient registration	●														
Patient background	●														
BFI		△						△							△
SBM		〇	〇	〇	〇	〇	〇	〇	〇	〇	〇	〇	〇	〇	〇
CSBM		〇	〇	〇	〇	〇	〇	〇	〇	〇	〇	〇	〇	〇	〇
Bowel movement, urgency, residual feeling, and shape of stool during defecation		〇	〇	〇	〇	〇	〇	〇	〇	〇	〇	〇	〇	〇	〇
Overall bowel movements in the past week		〇						〇							〇
Laxatives taken internally		〇	〇	〇	〇	〇	〇	〇	〇	〇	〇	〇	〇	〇	〇
Whether regular laxatives are started or changed								●							●
Number of times to use rescue laxatives		〇	〇	〇	〇	〇	〇	〇	〇	〇	〇	〇	〇	〇	〇
Number of times to use rescue painkillers		〇	〇	〇	〇	〇	〇	〇	〇	〇	〇	〇	〇	〇	〇
Vomiting frequency per day		〇	〇	〇											
Number of antiemetics used per day		〇	〇	〇											
PAC-QOL		〇													〇
PAC-SYM		〇													〇
EORTC QLQ-C15-PAL		〇						〇							〇
Vital signs	●														●
Cancer treatments administered during the protocol treatment period															●
Doses of opioids administered at regular intervals during the protocol treatment period															●
Adverse events during the protocol treatment period (CTCAE v5.0)															●
Responses to questions about the allocation groups															○
															●^a^

●: Health care provider evaluation.

△: Patient assessment (confirmed by blinded medical personnel).

○: Patient assessment.

BFI; Bowel Function Index, SBM; spontaneous bowel movement, CSBM; complete spontaneous bowel movement, PAC-QOL; Patient Assessment of Constipation Quality of Life questionnaire, PAC-SYM; Patient Assessment of Constipation Symptoms questionnaire, EORTC QLQ-C15-PAL; European Organization for Research and Treatment of Cancer Quality of Life Questionnaire Core 15 Palliative, CTCAE; Common Terminology Criteria for Adverse Events.

a The physician responsible for the Day 14 evaluation will respond.

### 2.5 Drug supply

Masking will be implemented by overencapsulation of naldemedine/placebo to produce identical capsules. Naldemedine will be supplied in capsules filled with the actual drug and lactose, and the placebo will be supplied in capsules filled with lactose. Naldemedine will be purchased from Shionogi & Co., Ltd. To improve adherence to interventional protocols, patients will be required to return the unused capsules at the last visit. Double-blindness will be verified by assessing the concordance between the assigned group as perceived by the patient and the physician responsible for the Day 14 evaluation and the group actually assigned.

### 2.6 Sample size calculation

As there are no interventional studies on the prevention of OIC in patients with cancer, enrollment was established from data of observational studies of OIC in patients with cancer and interventional studies on patients without cancer. A multicenter prospective observational study reported that 65% of patients with cancer who started regular opioid prescriptions and did not receive prophylactic laxatives developed OIC [2]. Other studies reported that the incidence of constipation in patients with cancer receiving opioids ranges from 63% to 81% [1,22,23].

On the other hand, the results of clinical trials in which naldemedine was administered prophylactically at the start of opioid therapy have not been reported. In a multicenter interventional study, approximately 70% of patients with cancer who were treated with naldemedine (Symproic® 0.2 mg) for OIC had three or more spontaneous bowel movements per week and an increase of one or more spontaneous bowel movements per week from baseline [7].

Assuming that the incidence of constipation is 35% in the naldemedine group and 65% in the control group, the significance level is 5% on two-sided tests, and the power is 80%, the required number of patients becomes 43 in each group, for a total of 86 patients. The target number of enrolled patients was set at 100 assuming a dropout rate of 10%.

### 2.7 Participation criteria

Patients who meet all of the following inclusion criteria at the time of enrollment will be eligible. The inclusion criteria are as follows: 1) Patients with cancer starting regular strong opioid (morphine, oxycodone, hydromorphone) medication for the first time for cancer pain, 2) age 20 years or older (at the time of obtaining consent), 3) patients who can take oral medications, meals, and beverages, 4) patients who are considered capable of self-documentation in the patient diary (proxy documentation in the patient diary is acceptable if the patient is capable of self-assessment), 5) patients who are not expected to experience a rapid change in their cancer condition during the protocol treatment period, and 6) patients who received sufficient explanation and have an understanding of the study, and who freely gave their written consent to participate.

The exclusion criteria are as follows: 1) Patients with gastrointestinal obstruction (actual or suspected) or patients with a history of gastrointestinal obstruction and a high risk of recurrence, 2) patients who have undergone surgery, radiotherapy, or procedures affecting gastrointestinal function (e.g., nerve blocks) within 14 previous days before the date of enrollment, or who will undergo such procedures within the protocol treatment period, 3) patients with medically significant cardiovascular, respiratory, hepatic, or renal dysfunction based on history, clinical laboratory values, electrocardiogram, or physical examination who are judged as inappropriate to participate in the study, 4) patients who previously took or are currently taking naldemedine, 5) patients who have severe diarrhea (more than 7 times a day) within 7 previous days before the date of enrollment or who underwent stool extraction for constipation, 6) patients who have used opioid patches or opioid injections within 7 previous days before the date of enrollment, 7) patients who are undergoing cancer drug therapy that is expected to affect defecation within 14 days (retrospectively) from the initial enrollment date or who are scheduled to undergo such therapy within the protocol treatment period. Cancer drug therapy that is expected to affect defecation is defined as follows: (1) First dose of a treatment regimen containing irinotecan and (2) other cancer drug therapy that is deemed certain to affect defecation, 8) pregnant or lactating patients, 9) patients with suspected hypersensitivity to opioid receptor antagonists, such as naldemedine, naltrexone, methylnaltrexone, naloxone, etc., and 10) other patients who are judged by the principal investigator or sub-investigator as inappropriate for participation in the study based on concomitant therapy or medical findings.

### 2.8 Randomization

The enrolled patients will be randomly assigned in a 1:1 ratio at the Data Center. Randomization will be performed after the patient has signed the informed consent form. To prevent significant bias in the random assignment of patients, a minimization method will be used with “institution,” “Eastern Cooperative Oncology Group Performance Status (less than or equal to 1, greater than or equal to 2),” “gastrointestinal cancer or non-gastrointestinal cancer,” and “regular laxative use (no or yes)” as assignment adjustment factors. As inter-institutional differences in the background of enrolled patients, treatment, efficacy evaluation, and safety evaluation are widely known, the institution will be set as an allocation-adjusted factor. Physical inactivity is a risk factor for the development of constipation; therefore, Performance Status will be set as an allocation factor [21]. In addition, as gastrointestinal cancer is likely to be a risk factor for constipation, gastrointestinal cancer or non-gastrointestinal cancer status will be used as an allocation factor. The presence of regular laxative use on the day before enrollment will be used as an index to evaluate the degree of constipation at the time of enrollment [4]. Information on the allocation groupings will be available only to the data management officer on the EDC system, and patients, other investigators, and statistical analysts will be kept blinded until unblinding.

### 2.9 Adverse event monitoring

For the evaluation of adverse events/adverse reactions, the Common Terminology Criteria for Adverse Events (CTCAE) v5.0 Japanese translation of Japan Clinical Oncology Group (http://www.jcog.jp/doctor/tool/ctcaev5.html) is used. Any of the following shall be considered a serious adverse event: 1) Deaths, including that occurring before the start of protocol treatment, 2) diseases that may lead to death, 3) CTCAE Grade 4 adverse events, 4) illness or other infirmities that require hospitalization or extension of the hospitalization period at a medical institution for treatment, 5) adverse events of CTCAE Grade 3 or lower that require hospitalization for more than 24 h or extended hospitalization for treatment of the adverse event, 6) disability: permanent or marked disability or dysfunction (excluding myelodysplastic syndrome, secondary cancer, etc.), or the threat of such disability, 7) diseases, etc. that may lead to disability (excluding myelodysplastic syndromes, secondary cancers, etc.) that cause or may cause permanent or significant disability or dysfunction, 8) illnesses, etc., which are serious in accordance with 1) to 5), and 9) congenital diseases or germ-line anomalies.

In the event of a serious adverse event that appears to be caused by the study drug, or in other cases where information on whether naldemedine was administered is considered to have a critical impact on the patient's prognosis or QOL, and it becomes necessary to clarify the details of the assigned group, the principal investigator and the Independent Data Monitoring Committee may judge that the relevant cases be unblinded and the content of the unblinding be communicated to the principal investigator.

### 2.10 Criteria for discontinuation

The protocol treatment will be discontinued in the following cases: 1) When protocol treatment cannot be continued due to adverse events, 2) if the patient requests discontinuation of the protocol treatment for reasons that cannot be ruled out in relation to the adverse event, 3) if the patient requests discontinuation of the protocol treatment for reasons that can be ruled out in relation to the adverse event, 4) death of a patient during protocol treatment, 5) in the event of rapid deterioration of the patient's condition after enrollment, discovery of protocol violation, or discovery of ineligibility, and 6) when stool extraction is performed.

### 2.11 Definition of protocol deviations

A protocol deviation is one in which the administration of a drug, clinical examination, or evaluation of toxicity or efficacy is not performed in accordance with the provisions of the protocol. At the time of monitoring, items suspected of possible deviations will be listed in the monitoring report as “possible deviations,” and will be classified into one of the following categories after review by the research secretariat and the research group: 1) Violation, 2) deviation, and 3) acceptable deviation.

### 2.12 Statistical analysis

The target population for analysis of efficacy will be a population of all randomized patients, excluding patients who meet at least one of the following criteria: 1) Patients who were found not to meet the eligibility criteria after randomization (post-hoc ineligible), 2) patients who have never received the protocol treatment, and 3) patients with no efficacy endpoints measured. The target population for analysis of safety will be a population of all randomized patients for whom the protocol treatment was administered at least once.

For the primary endpoint, the null hypothesis that the proportion of patients with a BFI of less than 28.8 on Day 14 in the naldemedine and placebo groups is equal will be tested at a two-sided significance level of 5%. A chi-square test will be used. Point estimates and 95% confidence intervals will be calculated for the proportion of patients with a BFI of less than 28.8 in each group and the difference in the proportion of patients with a BFI of less than 28.8 between the two groups. Patients who started or increased their doses of regular laxatives during protocol treatment will be treated as protocol deviations and evaluated by BFI on Day 14 (not excluded from the analysis). As a supplementary analysis, patients who started or increased their doses of regular laxatives during the protocol treatment period will be considered as treatment failure and will be treated as equivalent to patients with a BFI of 28.8 or higher at Day 14. If a significant number of deviations are observed, we will consider interpreting the results by exploratory analysis using the Day 7 results. An interim analysis will not be performed on the primary endpoint.

Of the secondary endpoints of efficacy, the proportion of patients will be evaluated similarly to the primary endpoint. BFI, PAC-QOL, PAC-SYM, each domain and each subscale score of EORTC QLQ-C15-PAL, and bowel movements (4-point scale) will be evaluated visually based on the calculation of summary statistics and trend plots of the mean and 95% confidence interval at each time point. The change in BFI from Day 1 to Day 7 and Day 14, and that in PAC-QOL and PAC-SYM from Day 1 to Day 14 will be compared between groups using a two-sample *t*-test, and the point estimates and 95% confidence intervals of the mean and difference between groups will be calculated. The number of spontaneous bowel movements, complete spontaneous bowel movements, use of rescue laxatives, and diarrhea episodes occurring during the protocol treatment period will be evaluated by group and time point.

For the endpoints of safety, the number and incidence of all adverse events occurring during the protocol treatment period, regardless of CTCAE Grade, will be calculated after grading using the Japan Clinical Oncology Group shared criteria range. In addition, the number and proportion of patients of CTCAE Grade 3 or higher and Grade 4 events will be calculated in the same manner. Missing, inadmissible, and abnormal data will be reviewed and confirmed by the principal investigator/Research Office and the Data Center prior to data fixation. The analysis will be performed using SAS, version 9.4 (SAS Institute, Cary, NC, USA).

### 2.13 Trial steering committee, data monitoring committee, and Audit Committee

The Protocol Review Committee/Independent Data Monitoring Committee/Audit Committee and the Data Center/Operating Office will be organized and operated by the Japanese Organisation for Research and Treatment of Cancer (JORTC), a non-profit organization that provides clinical research support for physician- and researcher-led clinical research, including support for the preparation of clinical research protocols, data management, and statistical analysis for quality control and quality assurance, preliminary evaluation of clinical research, and third-party monitoring and management systems to evaluate progress, safety, and effectiveness. This protocol was reviewed and approved by the Protocol Review Committee prior to submission to the Accredited Clinical Research Review Board. The Independent Data Monitoring Committee will also monitor the protocol during the study period. This study will be audited to ensure the reliability of the study results. An auditor nominated by the principal investigator and approved by the Chair of the Audit Committee will visit the facilities participating in the study to check the approval documents of the medical institutions, check patient consent documents, and compare the data entered in the EDC with the medical records in accordance with the audit plan prepared by the auditor and approved by the Chair of the Audit Committee.

### 2.14 Study flow

A flow chart of the study is shown in Fig. 1.

## 3 Discussion

This is the first study proposed to assess the superiority of naldemedine over placebo in the prevention of OIC through a placebo-controlled, double-blind, randomized controlled trial. It is unclear which laxatives are appropriate to prevent OIC; therefore, the control group was set to receive no such treatment. The BFI is the most commonly used index in the diagnosis of constipation, including OIC, based on previous studies [[24], [25], [26], [27]] and a multicenter observational study in Japan [2]. Thus, it was selected as the primary endpoint in this study because it can evaluate the preventative effects of naldemedine against OIC, which is the purpose of this study.

In this study, patients in both the naldemedine and placebo groups who are being treated for constipation using existing laxatives at the time of enrollment are to continue with no change in dosage or administration until the end of the study, but when there are no bowel movements for more than two days, the single use of another laxative is allowed to minimize patient discomfort. This two-day time frame was set in accordance with clinical studies on chronic constipation [28,29]. The protocol treatment period was set at 14 days as the shortest period for which effects have been observed based on previous studies and feasibility [2,30].

This trial may have the following limitations. First, the rationale for sample size estimation is weak because there have been no interventional studies on the prevention of OIC in patients with cancer. Second, the BFI for Days 7 and 14 may not always be evaluated in person, which may reduce its reliability.

## 4 Conclusions

If this study demonstrates that naldemedine is effective at opioid initiation in reducing the incidence of OIC in patients with cancer, clinical standards of care will be altered through changes in national and international guidelines. Thus, the frequency of OIC will be reduced, which will improve patient QOL and prevent the discontinuation or reduction of opioids due to side effects.

## Ethics approval and consent to participate

We obtained approval for this study from the Tsukuba University Clinical Research Review Board on January 26, 2021 (approval reference number TCRB20-001). Written informed consent for participation in the study will be obtained from all participating patients.

## Consent for publication

Written informed consent for publication will be obtained from all participating patients.

## Availability of data and materials

The datasets used and/or analyzed during the current study will be available from the corresponding author on reasonable request.

## Author contributions

JH was responsible for conceptualizing and developing the study protocol. TH wrote this manuscript. TK, SK, MS, TY, Y. Yamamoto, T. Moriwaki, and T. Morita provided essential feedback for the design of this study. AN and HN provided important revisions to this manuscript. M. Hirakawa and YH will recruit patients and conduct follow-up. KA and K. Kihara will allocate patients. SO will analyze data. Y. Yamanaka, K. Konishi, KD, YT, M. Horiuchi, and M. Homma will manage drug supply. All authors read and approved this manuscript in its current state.

## Funding

This study is sponsored by the 10.13039/501100006559University of Tsukuba: Grant for Implementation of Advanced Medicine (GIAM) and and funded by the Japanese Society of Palliative Medicine. This protocol was not reviewed by the funding body.

## Declaration of competing interest

The authors declare that they have no known competing financial interests or personal relationships that could have appeared to influence the work reported in this paper.

## Data Availability

No data was used for the research described in the article.

## List of Abbreviations

OIC: opioid-induced constipation
PAMORA: peripherally-acting μ-opioid receptor antagonist
EDC: electronic data capturing
QOL: quality of life
BFI: Bowel Function Index
PAC-QOL: Patient Assessment of Constipation Quality of Life questionnaire
PAC-SYM: Patient Assessment of Constipation Symptoms questionnaire
EORTC QLQ-C15-PAL: European Organization for Research and Treatment of Cancer Quality of Life Questionnaire Core 15 Palliative
CTCAE: Common Terminology Criteria for Adverse Events
